# Crosstalk between MicroRNA and Oxidative Stress in Physiology and Pathology 2.0

**DOI:** 10.3390/ijms23126831

**Published:** 2022-06-20

**Authors:** Antonella Fioravanti, Antonio Giordano, Francesco Dotta, Luigi Pirtoli

**Affiliations:** 1 Rheumatology Unit, Department of Medicine, Surgery and Neuroscience, Azienda Ospedaliera Universitaria Senese, Policlinico Le Scotte, 53100 Siena, Italy; 2 Sbarro Institute for Cancer Research and Molecular Medicine, Department of Biology, College of Science and Technology, Temple University, Philadelphia, PA 19122, USA; giordano@temple.edu (A.G.); luigipirtoli@gmail.com (L.P.); 3 Department of Medical Biotechnologies, University of Siena, 53100 Siena, Italy; 4 Diabetes Unit, Department of Medicine, Surgery and Neurosciences, University of Siena, Policlinico Le Scotte, 53100 Siena, Italy; francesco.dotta@unisi.it

MicroRNAs (miRNAs) are a class of small non-coding RNAs around 22 nucleotides long that regulate gene expression by binding specific sequences within target messenger RNA (mRNA). Growing evidence demonstrated their role in physiological cellular processes and in different pathological conditions, including cancer, cardiovascular diseases, diabetes mellitus, and neurological and rheumatic disorders. Furthermore, miRNAs are promising diagnostic and prognostic biomarkers, and potential therapeutic targets for different diseases.

The release of reactive oxygen species (ROS), such as superoxide anion, hydrogen peroxide (H_2_O_2_), and nitric oxide, derives from cellular oxidative metabolism and plays a pivotal role in many cellular functions. Under normal conditions, the production of endogenous ROS is balanced by the antioxidant defense system. ROS are essential for normal cellular processes, while their aberrant production, or the failure of the capacity to scavenge excessive ROS, determines an altered redox status with an excessive synthesis of free radicals, leading to an imbalance in the redox environment of the cell.

The loss of normal ROS levels can cause damage of lipids, proteins, and DNA, all of which contribute to the development of various pathologies as neurological disorders, rheumatic and cardiovascular diseases, diabetes, and cancer.

Recent evidence has shown a cross-talk between miRNAs and oxidative stress even if this complex and mutual interaction needs to be amply elucidated.

In fact, we hinted at the great complexity of this subject in the Editorial preceding the first special issue of International Journal of Molecular Sciences on cross-talk between oxidative stress and miRNAs. The valuable contributions included in the present update shed more light on many unresolved questions in this fields; however, much remains to be done for attempting to translate into clinics the reported disclosures. 

The new Special Issue entitled “Crosstalk between MicroRNA and Oxidative Stress in Physiology and Pathology 2.0” of the International Journal of Molecular Sciences includes a total of 21 contributions: 9 Original Articles and 12 Reviews providing new information about the interaction between miRNAs and oxidative stress under normal and pathological conditions. 

Grieco et al. [1] extensively reviewed the recent progress in the field about the role of miRNAs in the regulation of the expression of several factors causing or preventing oxidative stress in Type 1 and Type 2 diabetes or in diabetic complications. Indeed, the authors discuss the possible therapeutic interventions and new delivery strategies aimed at modulating miRNAs involved in diabetes.

The review performed by Filardi et al. [2] focused of non-coding RNAs (ncRNAs) in Gestational Diabetes Mellitus (GDM), referring new evidence on their role in the complex pathophysiological mechanisms of GDM. Promising data suggest a potential function of several circulating ncRNAs as early predictors of GDM. The remarkable stability in extracellular fluids and the easy collection from peripheral blood samples are the main strengths of these molecules as putative biomarkers for GDM, although current studies are still insufficient to draw firm conclusions and some inconsistencies have emerged.

A summary of the current knowledge regarding the interplay between miRNAs and oxidative stress in metabolic syndrome and its components was reported in the review by Włodarski et al. [3]. Collected data showed a very interesting link between miRNA and oxidative stress. The authors highlighted the relevance of miRNAs as therapeutic targets and potential biomarkers of disease progression.

Altered miRNAs levels have been associated with the production of ROS and mitochondrial dysfunction that occur in many neurodegenerative diseases such as Alzheimer’s, Parkinson’s, and Huntington’s disorders. The crosstalk existing among oxidative stress, mitochondrial dysfunction, and miRNAs dysregulation was the topic of the review performed by Catanesi et al. [4]. Based on the reported evidence, miRNAs seem to play essential roles in the regulation of genes involved in mitochondrial integrity and oxidative stress in different brain pathological conditions. However, the authors recommended caution in the interpretation of available results, mainly related to the experimental models (in vivo and in vitro) considered in different studies.

Gayen et al. [5] investigated the release of exosomal miRNAs secreted by human astrocytes under pro-inflammatory and oxidative stress conditions, and compared them to exosomal miRNAs released from resting astrocytes. For this purpose, human fetal astrocytes were stimulated with recombinant human interleukin (IL)-1β inducing the release of a specific subset of miRNAs via exosomes, which may have a potential role in regulating the inflammatory response. Additionally, these miRNAs may serve as potential biomarkers of neuroinflammation associated with neurological disorders and injuries.

Keuls et al. [6] addressed the subject of the possible involvement of miR-302 in coordinating the metabolic landscape of neural tube closure. MiR-302 family members are among the most highly expressed miRNAs in human embryonic stem cells and their deletion in mice results in a fully penetrant neural tube closure defect. In this original article, the authors used the miR-302 knockout mouse model demonstrating a link between glycolysis and ectodermal cell proliferation and an up-regulation of genes involved in glycolysis, including predicted miR-302 targets Pfkp, Pfkfb3, and Hk1. Taken together, the obtained results showed a critical role for miR-302 in the control of the glycolysis pathway and in the regulation of proliferation of the ectoderm during neural tube closure. 

Several in vitro studies highlighted a crosstalk between miRNAs, oxidative stress, and the role of hydrostatic pressure (HP) as modulators of morphology and metabolism of the cartilage cells. Interestingly, preliminary experiments on human chondrocyte cultures demonstrated the ability of HP to modify the expression levels of some miRNAs, involved in the pathogenesis of osteoarthritis, and to regulate oxidative stress balance. On the basis of this evidence, Cheleschi et al. [7] investigated the role of miR-34a, miR-146a, and miR-181a as possible mediators of HP regulation of oxidative stress in human osteoarthritic chondrocyte exposed to cycles of low sinusoidal and continuous HP. Furthermore, the use of miRNA-specific inhibitors confirmed the relationship among HP, the studied miRNAs, and oxidative stress, suggesting miRNAs as one of the mechanisms by which HP regulates chondrocyte metabolism and oxidative stress [7].

It has been reported that dairy cows can be susceptible to heat stress, which could result in a significant decrease in milk production and quality. Increasing evidence showed that circular RNAs (circRNAs), a type of ncRNA discovered in recent years, play an important role in the growth and development of animals, and that their biosynthesis was altered by heat stress. Wang et al. [8] demonstrated that the expression pattern of circRNAs in the mammary gland tissue of cows was markedly altered under heat stress. In the same study, the authors revealed that heat stress not only reduced milk yield but also affected quality, with a reduction in content of lactose and protein after increased temperature.

Haque et al. [9] thoroughly reviewed the role of miRNAs and oxidative stress in influenza A virus (IAV) pathogenesis. Some of the miRNAs directly regulate host immune responses while some modulate viral replication and gene expression. The characteristic relationship between miRNA expression and ROS generation contributes to IAV pathogenesis. Thus, the up- or down-regulation of miRNAs during IAV infections highlighted their potential as biomarkers for infection severity or pathogenesis.

The question whether miRNAs may be considered diagnostic markers and have possible therapeutic use in ischemic stroke was the leading argument of the review by Bulygin et al. [10]. They analyzed this subject as for the regulation of risk factors such as arterial hypertension, metabolic disorders, and atherosclerosis. To this regard, the pathophysiology of ischemic stroke was recapitulated, taking in account mechanisms like excitotoxicity, oxidative stress, inflammation, apoptosis, angiogenesis, neurogenesis, and Alzheimer’s disease, in which miRNAs can be involved. In this framework, these authors suggested that research should address the identification of possible miRNAs’ role for enabling risk evaluation, severity grade assessment, and suitable therapeutic management of ischemic stroke in the clinical setting.

Identifying oxidative stress-related genes as blood markers of coronary arterial disease has led Hueso et al. [11] to a complex and in-depth experimental approach addressing many putative miRNA-regulated oxidative stress genes in atherosclerosis progression. After compiling a list of 417 genes involved in the response to oxidative stress, they performed an integrated miRNA/mRNA counter-expression analysis, obtained a protein–protein interaction network and defined an expression profile in peripheral blood mononuclear cells (PBMCs) from patients affected by severe coronary disease as a consequence of atherosclerosis progression. This process allowed the identification of down-regulated FOXO1 and CCR7 as blood markers in this setting. The pathophysiology domain of oxidative stress-responsive miRNAs in heart injury was also the subject of the extensive review by Kura et al. [12], who considered changes in miRNAs expression occurring mainly through the modulation of nuclear factor erythroid 2-related factor 2 (Nrf2), sirtuins, calcineurin/nuclear factor of activated Tcell (NFAT), or nuclear factor kappa B (NF-kB) pathways. They outlined the role of several circulating ROS-related miRNAs both as possible biomarkers of various kinds of heart diseases, and in the perspective of therapeutic purposes, thus evidencing the necessity of further research.

The evaluation of circulating miRNAs as markers of mitral valve prolapse and regurgitation was the innovative subject of the study of Songia et al. [13], who showed a miRNAs signature associated with this condition. In order to discriminate the affected patients from healthy subjects, they used a Human MicroRNA Card A to examine plasma samples for miRNAs profiles, followed by real time PCR validation, and implemented a suitable machine learning analysis. miR-140-3p, 150-5p, 210-3p, 451a, and 487a-3p resulted as significantly up-regulated, whereas miR-223-3p, 323a-3p, 340-5p, and 361-5p were down-regulated, respectively, in affected and healthy subjects (*p* ≤ 0.01). More, the machine learning approach’s discriminating power allowed high accuracy (0.93) with a 0.97 AUC at the ROC curve. Possibly related biological processes were discussed. The investigated molecular signature is proposed as a suitable, first-line clinical test. The role of miRNAs in valvular heart diseases was also addressed by Nappi et al. [14]. They revised the available literature concerning stimulatory or inhibitory roles in mitral valve prolapse development, aortic leaflet fusion, and calcification pathways, etc. Selected pathologic entities, such as mitral prolapse and rupture of chordae tendineae were deemed suitable fields of investigation for assessing the related over- or under-expression of particular miRNAs, and the related regulation of intra- and extra cellular signaling. Possible therapeutic targeted approaches were discussed, including both miRNAs inhibitors or, instead, enhancers of miRNAs activity, considering also the technical problems which may emerge in their implementation and delivering strategies.

The review by Climent et al. [15] dealt with ROS-miRNAs crosstalk in the more extended domain of both cardiac and pulmonary diseases. Diseases of the heart (such as cardiac hypertrophy, heart failure, myocardial infarction, ischemia/reperfusion injury, and diabetic cardiomyopathy) and lung (idiopathic pulmonary fibrosis, acute lung injury/acute respiratory distress syndrome, asthma, chronic obstructive pulmonary disease, and lung cancer) were considered, allowing the indication of a role for miR-34a, miR-144, miR-421, miR-129, miR-181c, miR-16, miR-31, miR-155, miR-21, and miR-1/206 during oxidative stress in both heart and lung diseases, for an improved knowledge of these subjects of investigation.

Nenna et al. [16] remarked the limited scientific knowledge available regarding miRNAs’ role in heart tumors when compared with the great development of this research field in other neoplasms, thus grounding their revision particularly addressed to cardiac myxoma. They suggested that future investigations should be devoted to the complex and interconnected roles of miRNAs in modifying the expression of cardiac transcription factors (miR-335-5p); increasing cell cycle trigger factors (miR-126-3p); interfering with ceramide synthesis (miR-320a); inducing apoptosis (miR-634 and miR-122); suppressing production of interleukins (miR-217); and reducing cell proliferation (miR-218). In fact, interpreting the mechanistic control of miRNAs and the related signal pathways for cell overgrowth is presently of high interest for the therapeutic implications in heart myxoma and other cardiac tumors.

The field cancerization of the respiratory tract by tobacco smoke and other agents in the development and evolution of non-small lung cancer was the subject addressed by Pirlog et al. [17] in their review. Direct cell-to-cell and vesicle mechanisms were considered for interactions between the involved normal and tumor cells and tumor microenvironment (TME), consisting of proteins, ncRNAs, and miRNAs. These authors analyzed the role of miRNAs for macrophage polarization in TME and the field undergoing cancerization, thus indicating a domain of molecular players in which the possibility of modulating these molecular mechanisms might restore the immunogenic capacity of the TME, thus possibly enhancing the presently limited effectiveness of therapy.

Triple-negative breast cancer lacks therapeutic targets (i.e., hormonal and growth factor receptors) and has an unfavorable course due to early metastases and chemotherapy resistance. The hypothesis that these features—dependent on cancer stem cells and the epithelial-to-mesenchymal transition—can be enhanced by the chemotherapy stress itself was the subject of the experimental work of Li et al. [18]. In fact, cultured breast cancer stem cells—allowed to recover after chemotherapy exposition—showed an enhanced sphere-forming capacity and changed their morphology while expressing epithelial-to-mesenchymal gene expression; these authors identified six miRNAs as potential regulators of this process. However, the migratory capacity of these cells resulted as unaffected. 

Lerner et al. [19] devised an in vitro model in order to assess the role of extracellular vesicles (EV), containing some miRNAs together with other biomolecules, as mediators of protective molecular pathways against the oxidative stress involved in the pathophysiology of glaucoma. In fact, EV derived from the non-pigmented ciliary epithelium (NPCE), exposed to oxidative stress, exerts protective effects on the cells of the trabecular meshwork (TM), draining the aqueous humor of the eye. They could confirm through a suitable experimental approach that EV derived from oxidized NPCE cells significantly protected TM cells from direct oxidative stress, by evaluating Nrf2-Keap1 (a major oxidative stress pathway) and the Wnt pathway involved in primary open-angle glaucoma. EV derived from oxidized NPCE cells significantly attenuated the Wnt protein expression in TM cells and activated major antioxidant genes. The TM cells exposed to EV derived from oxidized NPCE cells exhibited significantly lower oxidative stress and higher super oxide dismutase and catalase activity compared with controls. The authors hypothesize that these disclosures may apply beyond the domain of the glaucoma’s pathophysiology, and possibly represent a common response communication for other tissues. The subject of crosstalk between miRNAs and oxidative stress in open-angle glaucoma was also reviewed by Tabak et al. [20], addressing the synthesis and deposition of extracellular matrix, regulated by chronic oxidative stress in the tissues involved in this pathology. In fact, they outlined the description of down-regulating activity of some miRNAs on pro-inflammatory and pro-fibrotic signaling pathways, including NF-kB, TGF-beta2, Wnt/beta-catenin, and PI3K/AKT. These authors also considered the subject of the elevated intra-ocular pressure due to increased outflow resistance, with reference to collagen degradation stimulated by some miRNAs that can prevent extracellular matrix deposition in the trabecular meshwork. Further, mitochondrial dysfunction due to oxidative stress can be suppressed by different miRNAs. In contrast, other miRNAs can inhibit the mTOR pathway, thus increasing oxidative damage. These authors concluded that specific miRNAs may be the subject of future research as promising therapeutic agents in primary open-angle glaucoma. 

Kiel et al. [21] reported the experimental evidence in an animal model that two miRNAs (mmu-mir-486a-5p and mmur-mir-92a-3p) are consistently dysregulated in retinal pigment epithelium and blood after laser-induced choroidal neovascularization. Further, in vitro experiments showed that these miRNAs significantly impacted microglial cell viability and mobility. The authors hypothesized possible therapeutic developments for complications of choroidal neovascularization based on these disclosures.

No papers proposed for this special issue primarily addressed translational works or clinical randomized trials. In fact, several related studies are registered at the National Institutes of health (https://clinicaltrials.gov, 6 June 2022) as other authors correctly commented without establishing, however, sound grounds for developing miRNA-based drugs. Indeed, the present academic research and industrial research and development processes seem to have taken different paths, following different interests in all fields of biomedical sciences. Unfortunately, the results of the preclinical investigations, made in house by industry and preceding the sponsored trials and marketing of new drugs, are not universally available, whereas the disclosures by independent institutions are not often deemed to deserve interest for prospective trials, when the necessary huge funding is taken into account. In this regard, we believe that the Scientific Societies should make a strenuous effort to involve politics and public authorities in order to resolve this dichotomy, which is proving to be counter-productive for medical research.
